# Exploring mitochondrial functions and dysfunctions in chondrocytes: toward the identification of novel therapies for osteoarthritis

**DOI:** 10.1038/s41413-026-00548-y

**Published:** 2026-06-01

**Authors:** Lucie Danet, Jérôme Guicheux, Marie-Astrid Boutet, Claire Vinatier

**Affiliations:** 1 Nantes Université, Oniris, CHU Nantes, INSERM, Regenerative Medicine and Skeleton, RMeS, UMR1229, F-44000 Nantes, France., Nantes, France; 2 Centre for Experimental Medicine & Rheumatology, William Harvey Research Institute and Barts and The London School of Medicine and Dentistry, Queen Mary University of London, London, UK

**Keywords:** Energy metabolism, Pathogenesis, Diseases

## Abstract

Mitochondria are essential organelles primarily described for their vital role in producing energy through oxidative phosphorylation (OxPhos). Due to the hypoxic environment of chondrocytes and their heavy reliance on glycolysis, mitochondrial functions have long been considered of minimal relevance in these cells. However, as major suppliers of energy through the ATP they produce by OxPhos, mitochondria help to regulate the balance between anabolism and catabolism. In osteoarthritis (OA), the most prevalent joint disease, this balance is dysregulated. In addition, correlations between metabolic disorders and the risk of developing OA are also increasingly studied. In this context, mitochondrial dysfunctions in OA chondrocytes are emerging as a relevant area to propose efficient, yet unavailable, disease-modifying OA drugs (DMOADs). This narrative review examines the underlying mechanisms by which the mitochondrial functions become dysregulated in chondrocytes during OA. Drawing on up-to-date literature, it highlights how both structural and functional alterations of mitochondria contribute to OA pathology in chondrocytes. Finally, this review discusses the potential of mitochondria-targeted therapeutic strategies for OA, framed within a conceptual “*repair* or *replace*” approach.

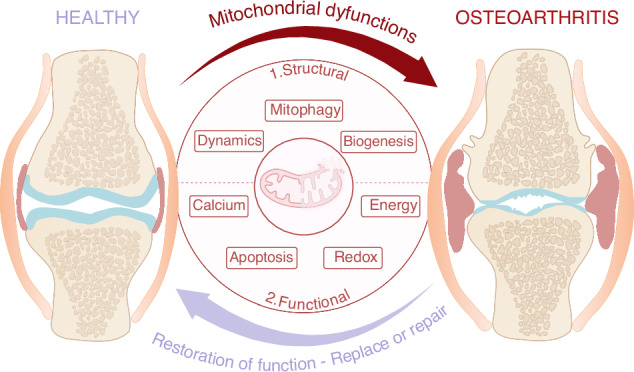

## Introduction

Mitochondria are double-membrane-enclosed organelles found in most eukaryotic cells. Over fifty years ago, the endosymbiotic theory proposed that mitochondria originated when a primitive eukaryotic cell engulfed a high-energy bacterium, thereby offering protection in exchange for energy advantage.^1^ Ultimately, mitochondria retained features of their ancestor, including their genome, a double-membrane structure, and internal cristae that increase the surface area of the inner mitochondrial membrane (IMM), the site of energy production.^2^ The mitochondrial respiratory chain (MRC) is integrated within the IMM, where oxidative phosphorylation (OxPhos) occurs in the presence of oxygen to produce adenosine triphosphate (ATP).^3^

In humans, most tissues receive oxygen through blood perfusion (physioxia) supporting aerobic pathways such as OxPhos.^4^ However, some tissues are poorly perfused and exist in a low-oxygen environment under physiological conditions (physiological hypoxia), relying solely on oxygen diffusion. Articular cartilage is one of the most prototypical examples of such tissue. It is an avascular connective tissue that covers the ends of bones in synovial joints, providing a smooth and lubricated surface that enables low-friction movement. Cartilage cells, namely chondrocytes, thus naturally reside in a low oxygen environment with oxygen tension ranging from approximately 10% at the surface to near anoxia in the deeper zone, decreasing with cartilage depth and distance from the synovial fluid.^5^ Overall, chondrocytes are well adapted to low oxygen conditions, allowing them to maintain their functions and support tissue homeostasis.^6^ They predominantly use glycolysis, an anaerobic metabolic pathway occurring in the cytosol, rather than OxPhos, which is aerobic and takes place in the mitochondria. In this context, chondrocytes have long been considered to have low metabolic activity and the research on their mitochondrial functions has been sparse.

One of the most prevalent diseases affecting the joints, and in particular the articular cartilage, is osteoarthritis (OA). OA impacts 500 million people worldwide and predominantly affects the knees, hands, and hips, but can also develop in any mobile joint.^7^ OA is influenced by multiple risk factors, including age, sex, obesity, genetics, environmental exposures, biological changes, and biomechanical stress and previous traumatic injuries.^8^ Age is a major risk factor, with the prevalence of knee OA increasing markedly after age 50 and reaching its maximum around age 80.^7^ Obesity and metabolic syndrome further contribute to the increasing incidence of OA. OA impairs all the joint tissues, and is characterized by bone remodeling,^9^ synovial and infrapatellar fat pad inflammation and fibrosis,^10^ cartilage degradation,^11^ meniscus damages^12^ and ligament degeneration.^13^ As a result, this condition leads to chronic and disabling pain, resulting in decreased mobility and various comorbidities. The management of OA patients typically includes physical exercise, dietary adjustments, pain relief through analgesics or non-steroidal anti-inflammatory drugs (NSAIDs), and intra-articular corticosteroid injections. In advanced stages of OA, total joint replacement by arthroplasty is often inevitable. Despite extensive research efforts, there is no treatment able to stop or even slow down OA progression.^14^ Consequently, OA represents a major economic burden for healthcare systems, estimated at up to 2.5% of gross national product in some economically developed countries such as the United States, the United Kingdom, or Australia.^15^ This underscores the urgent need for developing disease-modifying OA drugs (DMOADs).

To date, mitochondrial dysfunctions have mainly been described in OA chondrocytes, whereas data regarding other joint cell types, such as fibroblast-like synoviocytes (FLS) and bone cells, remain limited.^16,17^ Accordingly, this review mainly focuses on chondrocytes, for which mitochondrial dysfunctions in OA are best described. The present narrative review aims to provide a comprehensive summary of the current knowledge of mitochondrial functions and dysfunctions in healthy and OA chondrocytes, allowing exploration of novel therapeutic options for this debilitating condition within the conceptual framework to *replace* or *repair* mitochondria. Accordingly, the first section provides an up-to-date overview of mitochondrial functions in chondrocytes under physiological conditions, addressing both structural (biogenesis, dynamics, and mitophagy) and functional (calcium homeostasis, apoptosis regulation, redox state maintenance, and energy metabolism) aspects. The second section then explores how these mitochondrial functions become dysregulated in chondrocytes in the context of OA. Consequently, future therapeutic strategies are likely to develop along two complementary axes in the final section of this review: “*Replace*”, which involves either replacing the entire organelle or replacing a complete mitochondrial function, and “*Repair*”, which focuses on restoring specific features of mitochondrial functions.

## Mitochondria: a unique organelle with key functions in chondrocytes

A unique feature of mitochondria is that they possess their own genome, which plays a crucial role in their functions.^18^ This mitochondrial genome consists of a single chromosome of double-stranded DNA, often referred to as mtDNA, which is maternally inherited. The mtDNA encodes 37 genes, all exclusively dedicated to mitochondrial functions. These include 22 transfer RNA (tRNA), 2 ribosomal RNA (rRNA), and 13 protein-coding genes that are all essential components of the MRC. Nevertheless, only 1% of mitochondrial proteins originate from the mitochondrial genome,^19^ the remaining proteins are encoded by the nuclear genome and imported into mitochondria. This import is facilitated by specialized transporters called translocons of the outer membrane (TOM) and of the inner membrane (TIM).^20^ Consequently, both the nuclear and the mitochondrial genomes are essential for maintaining mitochondrial integrity and functions.

Although mitochondria are best known for their ability to produce energy, they have many other functions. In 2024, a well-documented and comprehensive literature review has already detailed these mitochondrial features. Based on these findings, we propose a classification into mitochondrial structural and functional aspects.^21^ The present section therefore, examines these mitochondrial features, focusing on their specificities in chondrocytes under physiological conditions, as detailed in Table 1. As in every other cell, structural features, such as mitochondrial dynamics, mitophagy, and biogenesis regulate the number of mitochondria in chondrocytes. While no precise range for mitochondrial number per cell has been reported in chondrocytes, they appear to be relatively few, contrasting with cell types known to contain high levels of mitochondria, such as hepatocytes (~2 500 mitochondria per cell)^22^ and cardiomyocytes (~5 000 mitochondria per cell).^23^

**Table 1:** Structural and functional features of chondrocyte mitochondria under physiological conditions

	Mitochondria feature	Roles	Mechanisms	Molecular players	References
Structural	Dynamics	•Encompass fusion and fission processes •Fusion drives the formation of elongated mitochondria: -To allow the exchange of metabolites and mtDNA -Much more energetic •Fission results in fragmented mitochondria: -To ensure their proper distribution in chondrocytes -Lower energy output	- Fusion processes are mainly under the control of OPA1 and MFN1/2 proteins. - Fission processes are under the control of DRP1 and FIS1 proteins.	- OPA 1 - MFN1/2 - DRP1 - FIS1	^185–187^
	Mitophagy	•Selective removal of defective mitochondria for lysosomal degradation: -Ensure a sufficient pool of functional mitochondria	- Loss of mitochondrial membrane potential (ΔΨm). - PINK1/Parkin activation and ubiquitination of OMM proteins (ubiquitin-based pathway). - Alternative involvement of FUNDC1 mitophagy within chondrocytes (receptor-based pathway).	- PINK1 - Parkin - FUNDC1 - SIRT3	^73,188^
	Biogenesis	•Synthesis of new mitochondria: -Ensure a sufficient pool of functional mitochondria	- PGC-1α activation either through p-AMPK-mediated phosphorylation or SIRT1-mediated deacetylation. - NRF1 and NRF2 transcription factors promote *TFAM* expression. - TFAM mitochondrial transcription factor upregulation (mtDNA packaging, replication and transcription).	- PGC-1α - NRF1/2 - TFAM - SIRT1	^189^
Functional	Calcium homeostasis	•Regulation of intracellular calcium levels by uptake and release of calcium by the mitochondria: -Involved in intracellular signaling -Involved in extracellular matrix (ECM) calcification	- Utilization of mitochondrial specialized calcium transporters (VDAC, MCU and NCX). - Mitochondrial calcium granules contribute to calcification (e.g., ECM at cartilage growth plate level).	- VDAC - MCU – NCX	^25–27^
	Intrinsic apoptosis	•Initiation of apoptosis by the mitochondria: -To allow the removal of damaged chondrocytes and help to protect cartilage	- Intracellular pro-apoptotic signals. - BAX and BAK proteins oligomerize at the OMM. - Pore formation and release of apoptotic factors in the cytosol (Cytochrome c, IMS mitochondrial proteins, mtDNA). - Apoptosome formation and caspases activation.	- BAX - BAK - Cytochrome c	^190,191^
	Redox state maintenance	•ROS production and antioxidant systems in the mitochondria: -mtROS production during OxPhos (Complex I and III) -Mitochondria have their own enzymatic ROS scavenging systems -mtROS regulate intracellular signaling related to inflammation and hypoxia	- 1%-2% of mitochondrial oxygen consumption generates mtROS through OxPhos. - SOD2, the main mitochondrial antioxidant enzyme, converts O2•- to H₂O₂. - PRX3 reduces H₂O₂ to H₂O. - GPX1 and GPX4 could also reduce H₂O₂ to H₂O.	- SOD2 - PRX3 - GPX1/4 - IDH2 - SIRT3	^192^
	Energy production	•Production of ATP at high yield through OxPhos: -ATP fuels anabolism, including ECM proteins synthesis -Prevent cartilage degradation	- Complex I: NADH from the TCA cycle donates electrons to Complex II (4 protons into the IMS) (see Fig. 1). - Complex II: FADH₂ donates electrons to CoQ, which transfers them between Complexes I, II, and III. - Complex III: Transfer of electrons to cytochrome c (4 protons into the IMS). - Complex IV: Transfer of electrons to oxygen, forming H_2_O (2 protons into the IMS). - Complex V (ATP synthase): Use of proton motive force to convert ADP and Pi into ATP.	- NADH - FADH2 - CoQ - Cytochrome c - Complexes I-V - ATP	^193^

ΔΨm mitochondrial membrane potential, *ADP* adenosine diphosphate, *ATP* adenosine triphosphate, *CoQ* coenzyme Q, *DRP1* dynamin-related protein 1, *ECM* extracellular matrix, *FADH*₂ flavin adenine dinucleotide, *FIS1* mitochondrial fission 1 protein, *FUNDC1* FUN14 domain containing 1, *GPX1* glutathione peroxidase 1, *GPX4* glutathione peroxidase 4, *IMS* intermembrane space, *MCU* mitochondrial calcium uniporter, *MFN* mitofusin, *mtROS* mitochondrial reactive oxygen species, *NADH* nicotinamide adenine dinucleotide, *NCX* sodium-calcium exchanger, *NRF1/2* nuclear respiratory factors 1 and 2, *OMM* outer mitochondrial membrane, *OPA1* optic atrophy 1, *OxPhos* oxidative phosphorylation, *Parkin* E3 ubiquitin-protein ligase Parkin, *Pi* inorganic phosphate, *PGC-1α* peroxisome proliferator-activated receptor gamma coactivator 1 alpha, *PINK1* PTEN-induced putative protein kinase, *PRX3* peroxiredoxin 3, *SOD2* superoxide dismutase 2, *TFAM* transcription factor A mitochondrial, *VDAC* voltage-dependent anion channel

Regarding mitochondrial functional features, calcium homeostasis is of particular interest in chondrocytes, given that the deep zone of articular cartilage is calcified. Like the endoplasmic reticulum, mitochondria act as calcium reservoirs in most cells. Intracellular calcium ions are transiently exchanged between mitochondria and the cytosol to maintain calcium homeostasis using specific transporters: the voltage-dependent anion channel (VDAC), the mitochondrial calcium uniporter (MCU) and the sodium-calcium exchanger (NCX).^24^ However, in contrast to other cell types, in growth plate chondrocytes, mitochondria do not merely function as calcium reservoirs, but rather accumulate calcium in the form of granules.^25^ Within chondrocytes from the growth plate, these granules can be transported from the mitochondria to the extracellular matrix (ECM), contributing to cartilage calcifications^26,27^ (Table 1). However, the presence of such granules in mature articular chondrocytes, outside of the growth plate, is currently unknown. While such mitochondrial calcium granules are also found in neurons,^28^ bone,^29^ and heart muscle cells,^30^ this phenomenon is not widespread across human cells, thus making their presence in chondrocytes noteworthy.

**Fig. 1:**
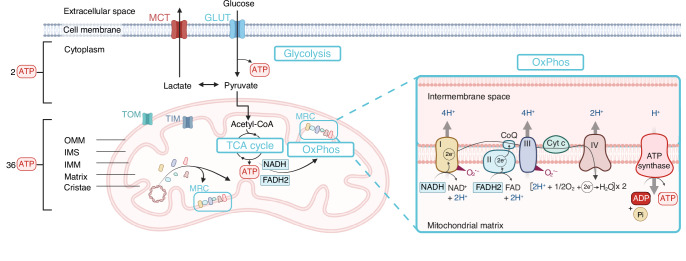
Involvement of mitochondria in chondrocytes energy metabolism through the engagement of the TCA cycle and OxPhos. Chondrocytes internalize glucose into the cell via GLUT transporters located on the cell membrane. Glucose is then metabolized by glycolysis to produce pyruvate. Pyruvate can be converted to either lactate or acetyl-CoA, the latter fueling the TCA cycle-OxPhos pathways. Zoomed area: NADH and FADH2, generated from the TCA cycle, feed electrons into Complex I and Complex II respectively. In the presence of oxygen, electrons are transferred along the different MRC complexes. This electron transfer pumps protons (H^+^) across the intermembrane space of the mitochondria, creating a proton gradient that drives ATP synthesis through ATP synthase. Abbreviations: ADP adenosine diphosphate, ATP adenosine triphosphate, CoQ coenzyme Q, CytC cytochrome c, FAD/FADH₂ flavin adenine dinucleotide (oxidized/reduced form), GLUT glucose transporter, IMM, inner mitochondrial membrane, IMS intermembrane space, MCT monocarboxylate transporters, MRC mitochondrial respiratory chain, NAD/NADH nicotinamide adenine dinucleotide (oxidized/reduced form), OMM outer mitochondrial membrane, OxPhos oxidative phosphorylation, Pi inorganic phosphate, TCA tricarboxylic acid, TIM translocase of the inner mitochondrial membrane, TOM translocase of the outer mitochondrial membrane

Finally, the most notable mitochondrial function is energy production along the MRC through OxPhos. It begins with the entry of glucose into the cell via the glucose transporters (GLUT), primarily GLUT1 in chondrocytes,^31^ followed by its conversion to pyruvate via the glycolysis in the cytosol (Fig. 1). Depending on the availability of oxygen, pyruvate can follow two possible pathways. In low-oxygen conditions, pyruvate is converted into lactate, and then effluxed from the cell through monocarboxylate transporters (MCT). Conversely, under normoxia, pyruvate is transported into the mitochondrial matrix and thus converted to acetyl-CoA. Subsequently, acetyl-CoA enters the tricarboxylic acid cycle (TCA cycle) to produce NADH and FADH2. These molecules both act as electron donors for complexes I and II of the MRC, thereby initiating OxPhos. The successive oxidation-reduction (redox) reactions generate a proton gradient that drives ATP production toward the final effector, ATP synthase, also known as complex V. Under physiological conditions, chondrocytes utilize both OxPhos (mitochondrial) and glycolysis (cytosolic) to produce energy, but at different rates. As mentioned, due to the low oxygen availability within the articular cartilage, chondrocytes heavily rely on glycolysis, accounting for approximately 75% of their ATP production, with the remaining ATP being produced by OxPhos.^32^ However, glycolysis yields significantly less ATP than OxPhos, producing 2 ATP per glucose molecule, compared to 36 generated through OxPhos. These various molecular components involved in chondrocyte energy metabolism, including glycolysis pathway, the TCA cycle and OxPhos are shown in Fig. 1. The structural and functional features of mitochondria are highly interrelated, as evidenced by mitochondrial dynamics that support energy function. Mitochondrial fission leads to a fragmented mitochondrial network and is generally associated with reduced ATP production, whereas fusion supports an interconnected mitochondrial network and enhanced energy output.^33^ Additionally, mitochondrial functional features are highly interconnected. For example, OxPhos dysfunction can lead to an increased formation of mitochondria reactive oxygen species (mtROS) and thus contribute to global oxidative stress. Indeed, an early reduction of oxygen can occur at complexes I and III levels due to strengthened electron leakage along the MRC.^34,35^ The structural and functional features of mitochondria in chondrocytes under physiological conditions are detailed in Table 1.

## Mitochondrial dysfunctions in OA chondrocytes

Impairment in various mitochondrial functions have been reported across a range of pathological conditions, including cancer,^36^ diabetes,^37^ obesity,^38^ chronic inflammatory diseases such as rheumatoid arthritis (RA),^39^ as well as age-related and neurodegenerative disorders such as Alzheimer’s^40^ and Parkinson’s diseases.^41^ In recent years, mitochondrial dysfunctions have been described in OA chondrocytes.^27,42^ The following section provides an overview of current research on mitochondrial dysfunctions in chondrocytes during OA.

### Association of mtDNA haplogroups and mutations with OA

The mitochondrial genome was the first mitochondrial feature extensively studied in OA. Throughout evolution, the mitochondrial genome has undergone mutations, resulting in the emergence of numerous common subgroups, termed mtDNA haplogroups.^43^ Both in vitro and in vivo studies have revealed correlations between specific mtDNA haplogroups and OA-related cellular processes. Cybrids, defined as cellular models that allow the incorporation of specific mtDNA haplogroups within a stable nuclear genome background,^44^ have been instrumental in establishing causal links between mtDNA and OA-like cellular phenotype, including increased oxidative stress and apoptosis.^45^ Interestingly, in vivo studies using conplastic mouse models, defined by identical nuclear genomes but different mtDNA, have shown modified OA progression profiles. These profiles depend on strain-specific mtDNA and have been corroborated in both post-traumatic OA (PTOA) model^46^ and age-related spontaneous OA model, thus highlighting the direct impact of mtDNA on OA susceptibility. The changes in OA progression were assessed by investigating the levels of cartilage degradation and synovitis in these models.^47^ Finally, in OA patients, mtDNA haplogroups J, T, and JT are linked to a reduced risk of developing OA,^48^ while haplogroups A, G, and H are associated with an increased risk.^49,50^ Beyond general haplogroups, specific single-nucleotide variants of mtDNA (mtSNVs) have also been implicated in OA susceptibility in humans. These variants are typically described by the “m” notation that indicates the position in the mitochondrial genome, followed by the reference and the mutated nucleotide when applicable. Several mtSNVs located in the hypervariable region of the D-loop of the mtDNA have been associated with differential OA risks. Variants such as m.16140 T > C and m.16217 T > C,^50^ confer protection against OA development, whereas m.16362 T > C^50^ and m.16519 C,^51^ are associated with an increased risk. m.16519 C variant, is associated with a reduced expression of the peroxisome proliferator-activated receptor gamma coactivator 1 alpha (PGC-1α), a key regulator of mitochondrial biogenesis, and with an increased oxidation of the Peroxiredoxin 3 (PRX3), leading to elevated mitochondrial superoxide anions levels. Moreover, gene ontology analysis of cybrids models harboring the m.16519 C variant revealed an upregulation of genes involved in acute inflammation, including Interleukin (IL)-6 (IL-6), directly connecting this mtSNVs to key OA pathological features.^52^ In addition, an accumulation of global mtDNA mutations or deletions,^53^ alongside a diminished mtDNA repair capacity, are described in primary human OA chondrocytes.^54^ Collectively, these data underscore the strong correlation between mtDNA variations and OA risk, thereby highlighting the critical role of mitochondrial integrity in the pathogenesis of OA.

### Variation of mitochondrial number in OA chondrocytes

To further elucidate mitochondrial perturbations in cartilage between physiological and OA pathological conditions, assessing differences in mitochondrial quantity may serve as a useful indicator. However, current quantification methods present significant limitations, often leading to conflicting findings.

The measurement of citrate synthase activity, the first enzyme in the TCA cycle, has been used as a marker to evaluate the number of mitochondria. Some studies report increased citrate synthase activity in OA chondrocytes compared to healthy controls,^55^ while others, particularly those using cybrid models, show reduced activity under osteoarthritic conditions.^44^ This discrepancy suggests that mitochondrial content may be modulated by extrinsic factors such as the cellular environment, age, and inflammatory stimuli.

Alternatively, mtDNA copy number quantification has also been used to estimate mitochondrial abundance, with one study reporting elevated mtDNA copy numbers in OA chondrocytes.^44^ Although mtDNA copy number may be a slightly more precise metric for counting mitochondria than the citrate synthase method, it is still an indirect measure that does not reflect the number of mitochondria, since each mitochondrion harbors multiple mtDNA copies, and this number can vary from one mitochondrion to another.

Overall, these inconsistent results emphasize the methodological challenge in accurately quantifying mitochondrial number: citrate synthase may better reflect the overall metabolic activity rather than the mitochondrial count, while mtDNA copy number, although informative, remains imprecise due to inherent variability in mtDNA content per organelle. To overcome the aforementioned limitations, transmission electron microscopy (TEM) may offer a precise means to identify and count mitochondria. However, only one study to date, including limited methodological details, has applied this approach and reported a reduced mitochondrial number in cultured primary human OA chondrocytes compared to healthy chondrocytes.^56^ Further investigations using TEM on human native cartilage-derived chondrocytes could validate these findings while avoiding artifacts introduced by cell isolation and culture. Nevertheless, it remains challenging to reliably capture mitochondrial mass, and the choice of technical approach must align with the study’s context.

**Fig. 2:**
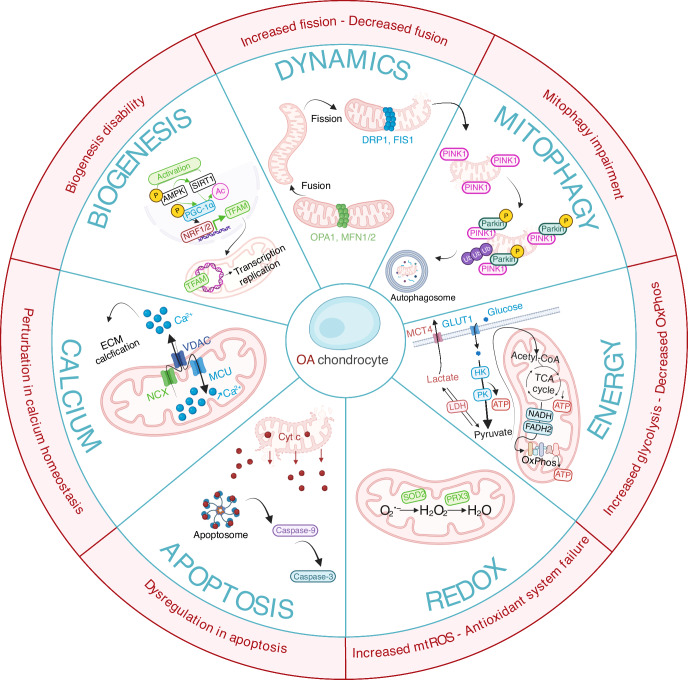
Mitochondrial functions and their alteration in OA chondrocytes. This schematic representation illustrates the functions of the healthy mitochondria and underlying key molecular mechanisms, and how they are altered in OA chondrocytes (in red). Mitochondrial functional features include calcium homeostasis, apoptosis, redox state maintenance and energy production. Mitochondrial structural features include mitochondrial biogenesis, mitochondrial dynamics and mitophagy. Abbreviations: AMPK AMP-activated protein kinase, ATP adenosine triphosphate, DRP1 dynamin-related protein 1, FADH2 flavin adenine dinucleotide, FIS1 fission protein 1, GLUT glucose transporter, HK hexokinase, LDH lactate dehydrogenase, MCU mitochondrial calcium uniporter, MCT monocarboxylate transporters, MFN1/2 mitofusin 1/2, NADH nicotinamide adenine dinucleotide, NCX sodium-calcium exchanger, OxPhos oxidative phosphorylation, OPA1 optic atrophy 1, PGC-1a peroxisome proliferator-activated receptor gamma coactivator 1-alpha, PK pyruvate kinase, PINK1 PTEN-induced kinase 1, PRX3 peroxiredoxin 3, SIRT1 sirtuin 1, SOD2 superoxide dismutase 2, TFAM mitochondrial transcription factor A, VDAC voltage-dependent anion channel

Beyond these observable features, several mitochondrial structural and functional processes within chondrocytes are altered during OA. The next section describes the relationship between these mitochondrial alterations and the development of the disease, illustrated in Fig. 2.

### Alterations of mitochondrial structural features in OA chondrocytes

#### Mitochondrial dynamics deficiency

Mitochondrial dynamics, the balance between fission and fusion processes that ensures optimal cellular function, is impaired in OA chondrocytes. Mitochondrial network fragmentation, governed by fission processes, has been observed in human OA chondrocytes and is further exacerbated by inflammation, which is inherent to the OA joint environment.^57^ In murine OA cartilage, this fragmentation has been associated with increased expression of the active form of the dynamin-related protein (DRP1) fission protein.^58–60^ Overall, DRP1 overexpression contributes to mitochondrial network fragmentation in OA chondrocytes, leading to reduced ATP production, increased oxidative stress,^33^ and ultimately exacerbating the OA phenotype. Conversely, the level of other fission proteins, such as mitochondrial fission 1 protein (FIS1), is diminished in primary human OA chondrocytes, thus highlighting the complex and still poorly understood interplay between molecular players and mechanisms regulating fission.^61^

Regarding fusion, the key proteins implicated are optic atrophy 1 protein (OPA1) and mitofusins 1 and 2 (MFN1/2) (Fig. 2). Among them, MFN2 displays increased levels in OA chondrocytes both in vitro, in primary rat and human OA chondrocytes, and in vivo in PTOA rat models, exerting pro-inflammatory effects through NF-κB and p38 MAPK pathways activation.^62,63^ In the same studies, silencing *MFN2*, either through RNA interference (siRNA) in vitro or via an in vivo knockdown approach, alleviates these harmful effects and slows down OA progression. Although *MFN2* overexpression seems paradoxical given the prevalent mitochondrial fragmentation in OA chondrocytes, it may reflect a compensatory mechanism to counterbalance the excessive fission activity. In addition, due to its strong association with inflammation, targeting *MFN2* expression could represent a potential therapeutic avenue. As for OPA1 fusion protein, a recent study highlighted that cartilage-specific deletion of *Opa1* in mice both impacts mitochondrial morphology and the mitophagic and autophagic pathways, and thus contributes to severe OA development.^64^ In addition to mediating mitochondrial fusion, OPA1 may also be involved in maintaining mitochondrial cristae integrity and regulating apoptosis.^65^ The exacerbated OA phenotype observed in *Opa1*-deficient cartilage mice likely results from subsequent mitochondrial dysfunctions.

Taken together, while the precise dynamic and regulatory interplay of fission and fusion proteins in OA chondrocytes remains to be fully deciphered, growing evidence supports their central role in preserving cartilage homeostasis. Importantly, mitochondrial dynamics are also closely linked to key mitochondrial processes such as mitophagy.

#### Mitophagy impairment

Mitophagy, an autophagy-based process targeting mitochondria for degradation, is compromised in OA. Recent studies have shown that both ubiquitin-mediated and receptor-based mitophagy are altered in OA cartilage. Notably, Parkin, an E3 ubiquitin ligase and a pivotal component of the ubiquitin-mediated mitophagy, is upregulated in primary human OA chondrocytes upon IL-1β stimulation.^66^ In this context, silencing Parkin through a *PRKN* siRNA exacerbates hallmark features of the IL-1β response, including ROS accumulation, mitochondrial membrane depolarization, and damage.^66^ Together, these in vitro findings indicate the inflammation-induced upregulation of Parkin in OA chondrocytes represents an adaptive, compensatory mechanism that confers mitochondrial protection.

Interestingly, the adaptive regulation of Parkin in OA chondrocytes may be partially mediated by sirtuin 3 (SIRT3), a mitochondrial deacetylase. SIRT3 has been shown to deacetylate Parkin, thereby modulating its activity,^67^ and may also indirectly regulate *Parkin* transcription via FOXO3, as demonstrated in osteoblasts.^68^ However, *SIRT3* expression is reduced in OA chondrocytes,^69,70^ which would be expected to result in reduced Parkin expression and activity. This is in clear contrast with the upregulation of Parkin observed in OA chondrocytes in vitro following IL-1β stimulation. These discrepancies underscore the limitations of acute inflammatory in vitro models and highlight the need to investigate these regulatory mechanisms in more complex ex vivo and in vivo pathological settings.

Consistently, in vivo studies reveal a more nuanced and, in some respects, opposing scenario. Indeed, *Prkn* knockout (KO) mice develop less severe OA compared with wild-type controls.^71^ This is evidenced by lower Osteoarthritis Research Society International (OARSI) scores, a semi-quantitative histological grading system that associates cartilage damage with OA progression. Similarly, PINK1, the mitochondrial kinase that recruits Parkin to depolarize mitochondria, has also been linked to OA. In a chemically-induced OA mouse model, PINK1 deficiency results in reduced cartilage degeneration and pain sensitivity as evidenced by a higher mechanical pain threshold in the von Frey test and a greater level of safranin-O staining in cartilage sections compared to wild-type mice.^72^ The authors proposed that the observed protection may be driven by a metabolic shift toward glycolysis in chondrocytes, which they interpret as a reversion to a younger cellular phenotype. However, the majority of studies instead associated an enhanced glycolysis with pathological reprogramming of OA chondrocytes, a concept that will be discussed in more detail later in section “Shift in energy metabolism” of this review.

Alternatively, we propose that the total invalidation of Parkin through *Prkn* KO, resulting in the inhibition of PINK1/Parkin-mediated mitophagy, may be beneficial in OA development, possibly by facilitating the engagement of alternative and more balanced mitophagy pathways. One such alternative is the receptor-mediated FUNDC1 pathway, which has recently gained attention in the context of OA. FUNDC1, which is located at the OMM, becomes active upon dephosphorylation and directly recruits LC3 to initiate mitophagy. Notably, both *FUNDC1* and *LC3B* expression are reduced in primary human OA chondrocytes as well as in the articular cartilage of mice with PTOA,^73^ suggesting impaired receptor-mediated mitophagy in disease progression. Importantly, pharmacological activation of FUNDC1-mediated mitophagy using KD025 (belumosudil), an FDA-approved ROCK2 inhibitor, has been shown to alleviate cartilage degradation in OA mouse models.^73^ Together, these findings suggest that OA progression may be driven not simply by defective mitophagy per se, but by an imbalance between PINK1/Parkin-dependent and receptor-mediated mitophagy pathways, with FUNDC1 playing a central protective role.

Additionally, these apparently contradictory results between in vitro and in vivo studies may reflect the dual role of mitophagy in OA. In the short-term, mitophagy may serve a protective function by clearing damaged mitochondria (e.g., acute inflammatory stress in vivo or IL-1β stimulation in vitro). However, a chronic activation of the PINK1/Parkin pathway in vivo could become detrimental, potentially interfering with cellular homeostasis. Despite these advances, the discrepancies between in vitro and in vivo studies, particularly regarding the PINK1/Parkin pathway, remain unresolved. Furthermore, the relative contributions of ubiquitin-mediated versus receptor-mediated mitophagy in the impaired mitophagy observed in OA chondrocytes require further investigation. It is also important to consider that mitophagy depends on the proper functioning of the general autophagic machinery, which is itself compromised in OA chondrocytes,^74^ suggesting that impaired autophagic flux may lead to the accumulation of dysfunctional mitochondria and contribute to disease progression.

#### Mitochondrial biogenesis disability

To compensate for mitochondrial dysfunction, chondrocytes can initiate mitochondrial biogenesis, a process that promotes the synthesis of new mitochondria. This mechanism is primarily regulated by PGC-1α, the master regulatory protein of mitochondrial biogenesis, which can be activated either by phosphorylated adenosine monophosphate-activated protein kinase (p-AMPK)-mediated phosphorylation or sirtuin 1 (SIRT1)-mediated deacetylation (Fig. 2). In human cartilage, the expression levels of AMPK and its active phosphorylated form p-AMPK both decline progressively with increasing OA severity.^75^ A similar trend is observed in the cartilage from both age-related and PTOA mouse models.^76^ AMPK deficient mice (*Prkaa* KO) exhibit accelerated OA progression and severity, characterized by higher OARSI scores, increased osteophyte formation, and exacerbated synovitis.^77^ This phenotype is further associated with elevated expression of catabolic enzymes such as matrix metalloproteinase-13 (MMP13).^77^ Notably, activation of the AMPK-PGC-1α signaling axis has been shown to counteract the deleterious effects of IL-1β. Moreover, this pathway has been proposed as a potential therapeutic target for alleviating pain in chemically-induced OA rat models.^78^ Indeed, the overexpression of the Sestrin-2 protein, an upstream activator of the AMPK-PGC-1α pathway, has demonstrated analgesic effects that are specifically mediated through AMPK activation, as these effects are abolished by an AMPK inhibitor.^78^

In parallel, SIRT1 which also activates PGC-1α through deacetylation, exhibits an inverse correlation with OA severity.^79–82^ Several studies have shown that *SIRT1* silencing exacerbates IL-1β-induced catabolic response in vitro^81^ and promotes OA progression in PTOA mouse models.^83^ In contrast, *SIRT1* overexpression in knock-in (KI) mice delays PTOA progression.^84^ Beyond its role in promoting mitochondrial biogenesis through PGC-1α deacetylation, SIRT1 also exerts protective effects by reducing inflammation and catabolism while supporting cell survival through the deacetylation of other target proteins, such as RelA/p65 for the NF-κB pathway and p53 for the regulation of apoptosis.^85,86^ In this context, a model combining SIRT1 activation with the selective inhibition of its deacetylation downstream targets (e.g., PGC-1α or RelA/p65) will be required to determine the relative contribution of each pathway to the protective effects of SIRT1 in OA progression. Therefore, the beneficial effects observed in *SIRT1* KI mice cannot be attributed solely to mitochondrial-dependent mechanisms, but may instead result from a synergistic combination of SIRT1 functions.

Consistent with these findings, key mitochondrial biogenesis regulators such as p-AMPK, SIRT1, PGC1-α and the transcription factors NRF1, NRF2, along with mitochondrial transcription factor A (TFAM), which act as ultimate effectors, are downregulated in primary human OA chondrocytes.^79^ Overall, these data suggest a failure of mitochondrial biogenesis in OA chondrocytes. Consequently, OA chondrocytes accumulate dysfunctional mitochondria that cannot be adequately removed via mitophagy nor replaced by de novo synthesis, contributing to progressive cellular dysfunction.

### Alterations of mitochondrial functional features in OA chondrocytes

#### Perturbation of calcium homeostasis

Calcium is an important player, not only in chondrocytes signaling through a direct activation of signaling pathways or an indirect role as a second messenger. In addition to its role in signaling, calcium handling by mitochondria may have implications for cartilage pathology. To date, the role of mitochondrial calcium in OA chondrocytes remains largely unexplored. Given that cartilage ECM calcification is a hallmark of OA,^87^ it has been hypothesized that mitochondria-derived calcium granules may contribute to this process by releasing excess calcium into the cartilage ECM.^88^ Mitochondria may also play a more active role in crystal formation through processes such as mitophagy and apoptosis, which can facilitate the nucleation of calcium-containing crystal precursors.^89^ It is therefore plausible that pathological crystals, such as basic calcium phosphate (BCP) or calcium pyrophosphate dihydrate (CCP), originate, at least in part, from chondrocytes mitochondria.^89^ Indeed, during mitophagy, damaged mitochondria are degraded in the acidic environment of the autolysosome, a process that has been described to lead to the formation of large amorphous calcium phosphate (ACP) deposits, released into the ECM by exocytosis.^90^ The hypothesis is supported by experiments showing that mimicking mitochondrial respiratory failure in chondrocytes can promote ECM mineralization, likely due to increased mitochondrial damage and mitophagy activation.^91^ Further research is needed to establish causal link between mitochondria calcium granules, mitophagy and cartilage ECM calcification during OA.

Importantly, mitochondrial calcium has essential functions beyond its potential involvement in the ECM mineralization. It is a key regulator of mitochondrial metabolism, stimulating several dehydrogenases enzymes within the TCA cycle and thereby enhancing ATP production through OxPhos.^92,93^ Moreover, calcium overload within mitochondria can trigger the opening of the mitochondrial permeability transition pore (mPTP), a critical event in the initiation of mitochondria-dependent apoptosis.^94^ Thus, mitochondrial calcium not only holds the potential to drive ECM calcification in OA but also fundamentally influences mitochondrial bioenergetics and cell fate in chondrocytes. A deeper understanding of the regulation and dysfunction of calcium signaling is essential to elucidate its multifaceted roles in OA pathogenesis.

#### Dysregulation in mitochondria-mediated apoptosis

Mitochondria also play a key role in mediating apoptosis, a function that appears to be altered during OA. In OA cartilage, the prevalence of apoptotic chondrocytes is markedly elevated, ranging from 18% to 21% of total cells, compared to 2%–5% in healthy cartilage.^95^ Apoptosis contributes directly to chondrocyte loss and is closely associated with the degradation of the cartilage ECM.^96^ This process is further exacerbated by elevated levels of ROS and pro-inflammatory cytokines in the OA joint environment.^97,98^ Mitochondrial membrane potential (ΔΨm) is known to decline with age in various cell types,^99^ a change that may predispose chondrocytes to apoptosis by facilitating mPTP opening and cytochrome c release. Such age-related mitochondrial alteration could therefore contribute to the increased apoptotic susceptibility observed in OA chondrocytes. Evidence for a pathological mitochondria-intrinsic pro-apoptotic program comes from OA cybrid models, which contain mtDNA from OA patients and exhibit an increased susceptibility to apoptosis compared to those containing mtDNA from healthy individuals.^100^ This study underscores that mitochondrial dysfunction, specifically driven by intrinsic mtDNA, may directly sensitize chondrocytes to apoptosis in OA, beyond the contribution of other extrinsic factors such as aging, oxidative stress, and inflammation.

#### Disruption in redox state maintenance

Oxidative stress is a hallmark of OA chondrocytes and arises from an imbalance between ROS production and cellular antioxidant capacity. Among these systems, mitochondria play a central role. In OA chondrocytes, the activity of the MRC complexes is impaired (see next section “Shift in energy metabolism”) leading to elevated production of superoxide anion (O_2_•^−^), a primary form mtROS (Fig. 2).^55^ A positive correlation between mtROS levels and OA severity has been documented in cartilage, with OA-derived cybrids exhibiting significantly higher mtROS levels compared to healthy ones.^44,101^ Consequently, this mitochondrial dysfunction contributed to the overall elevation in cellular ROS observed in OA chondrocytes. Such elevated mtROS inflict damage on mtDNA, which is particularly vulnerable due to its lack of protective histones and limited repair capacity. ROS-induced mtDNA damage further compromises MRC complexes function and OxPhos activity, leading to even greater mtROS production, thereby creating a self-perpetuating cycle of oxidative injury.^54^

In addition to elevated mtROS production, OA chondrocytes show a significant failure of their mitochondrial antioxidant defense systems. In human OA cartilage, the expression of the superoxide dismutase 2 (SOD2), also known as manganese-dependent superoxide dismutase (MnSOD), the primary mitochondrial antioxidant enzyme responsible for scavenging mtROS, is decreased.^102^ Consistently, cartilage-specific deletion of *Sod2* in vivo accelerates cartilage degradation in PTOA mouse models, likely as a consequence of excessive ROS accumulation, increased oxidative stress, and associated inflammatory responses, thereby highlighting the protective role of SOD2 in cartilage homeostasis.^103^ Similarly, the overexpression of PRX3, another mitochondrial antioxidant enzyme, has been linked to reduced OA severity in age-related mouse models, potentially by limiting H₂O₂ accumulation and thereby helping to protect mitochondrial membrane integrity.^104^ Additional mitochondrial antioxidant systems are also disrupted. The expression of *GPX4*, a key enzyme that belongs to the glutathione antioxidant system (GSH), is reduced both in IL-1β stimulated C28/I2 chondrocyte cultures and in the knee cartilage of a chemically-induced rat model of OA.^105^ In murine chondrocytes, mtROS contribute not only to mitochondrial dysfunction but also to a broader oxidative stress response affecting the entire cell, which is further amplified by the pro-inflammatory OA environment in chondrocytes.^106^ In addition, transcription factors specifically involved in managing oxidative stress and reducing ROS, such as NRF1/2, are downregulated in OA chondrocytes. Notably, SIRT3, often considered as an integral component of mitochondrial antioxidant defense due to its role in deacetylation and activation of SOD2 and IDH2,^107,108^ is progressively downregulated with OA severity in both human and murine cartilage.^70^ These reduced levels of SIRT3 may, at least in part, contribute to the widespread mitochondrial and redox imbalance observed in OA.

While a growing body of evidence supports a major role for global oxidative stress in OA pathogenesis, and has even led to clinical investigations of systemic antioxidants (e.g., NCT03350568 for dietary antioxidants), results remain inconclusive, and the therapeutic efficacy of broad-spectrum antioxidants oxidative stress modulation for OA still needs to be demonstrated. These limitations underscore the need for more targeted approaches that specifically address mitochondrial oxidative stress. While mitochondria-targeted antioxidants such as MitoQ have entered clinical trials for neurodegenerative (NCT00329056) and aged-related vascular dysfunction (NCT02597023), and SkQ1 (Visomitin) (NCT02121301) has received regulatory approval for dry eye disease, no mitochondria-specific antioxidants have yet been developed or approved for OA.

#### Shift in energy metabolism

As the primary site of ATP production via OxPhos, mitochondria are central to cellular energy metabolism, a process that is critically dependent on oxygen availability. While inflammatory cells may reduce local oxygen tension through increased metabolic demand, as hypothesized in the context of RA,^109^ oxygen availability in OA cartilage is instead generally described as increased. Indeed, pathological cartilage neovascularization, together with cartilage thinning during OA, also driven by aging and mechanical wear, can enhance oxygen diffusion within the tissue.^110,111^ Under such conditions of elevated oxygen availability, chondrocytes would be expected to enhance mitochondrial respiration and shift toward aerobic OxPhos for ATP production. However, paradoxically, OA chondrocytes undergo a counterintuitive metabolic adaptation that favors glycolysis over OxPhos, even in the presence of sufficient oxygen. This metabolic phenomenon, referred to as the Warburg effect or aerobic glycolysis was initially described in cancer cells but is increasingly recognized as a feature of OA chondrocytes, contributing to a shift in their functional metabolic state.^112^ This metabolic shift toward glycolysis is supported by increased levels of glycolysis-related metabolites, such as pyruvate and lactate, that have been detected in the synovial fluid of human,^113^ dogs^114,115^, and horses^116^ OA patients. PTOA sheep models also recapitulate this glycolytic phenotype.^117^ At the molecular level, this shifts is accompanied by aberrant upregulation of key glycolytic enzymes, such as hexokinase 2 (HK2),^42^ pyruvate kinase M2 (PKM2)^118^ and lactate dehydrogenase (LDHA).^119^

The increased reliance on glycolysis may initially represent an adaptive response of chondrocytes to cellular stress such as oxidative stress and inflammation, enabling chondrocytes to maintain ATP levels and sustain essential functions. However, when persisting chronically, this metabolic adaptation may become maladaptive and contribute to disease progression. In this context, an emerging hypothesis proposes that chronic glycolytic activation in OA chondrocytes occurs alongside dysfunctional OxPhos rather than representing a simple metabolic switch.

Indeed, alteration of OxPhos activity have been consistently reported in OA chondrocytes. Early studies identified a significant reduction in the activity of MRC complexes II and III.^55^ Subsequent work extended these findings, revealing additional impairment in the activity and expression of complexes I, IV, and V (ATP synthase) in OA chondrocytes.^79^ This OxPhos dysfunction is thought to originate, at least in part, from disrupted MRC complex biogenesis due to mtDNA damage, which may be driven by elevated ROS levels. The relative contributions of glycolysis and OxPhos to chondrocytes energy production may vary depending on disease stage and cellular phenotype, including hypertrophic or senescence states. Supporting this notion, cellular senescence is associated with a distinct form of mitochondrial dysfunction, known as senescence-associated mitochondrial dysfunction (SAMD), characterized by an impaired OxPhos and a compensatory shift toward glycolysis. In line with this, silencing the glycolytic enzyme *PKM2* has been shown to delay senescence progression in OA chondrocytes, as evidenced by reduced p16INK4a levels, decreased SA β-galactosidase activity, and increased *COL2A1* expression.^120^ Conversely, enhanced glycolytic flux can predispose chondrocytes to senescence in a PKM2-dependent manner, thus highlighting the tight interplay between energy metabolism and chondrocyte senescence.

Despite these advances, it remains unclear whether the observed shift toward glycolysis is a direct consequence of OxPhos dysfunction or whether it arises independently through inflammatory or stress-related signaling pathways. Moreover, the extent to which similar metabolic reprogramming occurs across distinct chondrocyte subpopulations during OA progression remains to be determined. Interestingly, pro-inflammatory cytokines such as tumor necrosis factor (TNF-α) and IL-1β can also exacerbate this glycolytic shift specifically in primary human OA chondrocytes, whereas healthy chondrocytes appear relatively resistant to this effect.^121^ This metabolic reprogramming, reduces ATP production and enhances catabolic activity, as evidenced by the upregulation of *MMP1*, *3*, and *13* relative expression.^122^

Consequently, OA chondrocytes globally become increasingly reliant on glycolysis at the expense of OxPhos, leading to inefficient energy production and promoting a cellular environment that favors catabolism over anabolism.^42,55^ The intricate relationship between inflammation, mitochondrial dysfunction, and altered energy metabolism underscores the therapeutic potential of targeting chondrocyte metabolic pathway in OA. Unlike conventional anti-inflammatory treatments, which primarily alleviate symptoms without addressing disease causes, metabolic intervention may directly correct the energetic and biosynthetic imbalances driving disease progression. This metabolic-based approach gains further relevance considering that other joint resident cells, such as FLS, also exhibit altered energy metabolism.

As an emerging hypothesis, considering the intense intercellular crosstalk within the joint, mitochondria or mitochondria-derived components may be exchanged between cells belonging to different tissues of the joint, potentially propagating mitochondrial dysfunctions. Supporting this concept, intercellular mitochondrial transfer from osteocytes to chondrocytes has been recently reported and shown to induce mitochondrial impairment in OA chondrocytes.^123^ In addition, it is conceivable that extracellular vesicles released by OA chondrocytes could reach neighboring joint cells and contribute to the initiation or spread of mitochondrial alterations. Together, these mechanisms represent promising but largely unexplored avenues for future research.

## Addressing mitochondrial dysfunctions in OA chondrocytes: *replace or repair*?

As discussed in previous sections, there is growing evidence of pathological mitochondrial alterations in OA chondrocytes, which may represent relevant therapeutic targets. Regenerative medicine, which seeks to restore the structure and function of damaged tissues, is particularly applicable to cartilage in the context of OA. Targeting one of those mitochondrial dysfunctions in OA chondrocytes, to re-establish cellular homeostasis and preserve cartilage integrity, fully align with regenerative medicine principles.

**Fig. 3:**
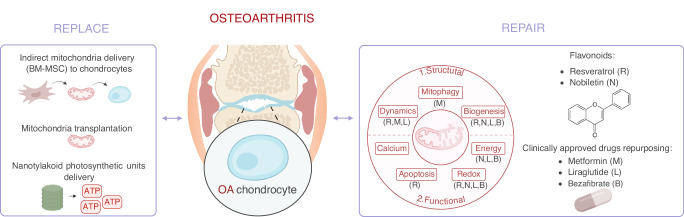
Targeting mitochondrial dysfunctions in OA chondrocytes: replace and repair-orientated therapeutic approaches. Replace strategies involve substituting dysfunctional mitochondria with a new pool of healthy mitochondria, either by indirect delivery through cell therapy or direct mitochondrial transplantation. This strategy also includes replacing a specific mitochondrial function, such as energy metabolism, through the delivery of nanotylakoid photosynthetic units. Repair strategies aim to improve mitochondrial function, for example through the use of flavonoids (e.g., resveratrol and nobiletin) and the repurposing of clinically approved drugs (e.g., metformin, liraglutide, and bezafibrate). The effect of each molecule on mitochondrial functions is indicated by the first letter of the molecule. Abbreviations: B bezafibrate, L liraglutide, M metformin, N nobiletin, R resveratrol

Among these strategies, we propose a classification along a “*replace or repair*” mitochondrial framework. Some of them are discussed in this section, in parallel with novel suggested therapies, and are illustrated in Fig. 3.

### Mitochondrial *replace* strategies

#### Stem-cell therapy and mitochondria transplantation

In light of the evident alterations in mitochondrial functions in OA chondrocytes, the therapeutic delivery of healthy mitochondria has emerged as a promising therapeutic strategy.^124,125^ The discovery that cells can engage in intercellular mitochondrial transfer has led to the development of indirect mitochondrial delivery approaches within the framework of cell therapy. For instance, bone marrow-derived mesenchymal stem cells (BM-MSC) have been shown to spontaneously transfer mitochondria to equine chondrocytes under stress conditions^126^ as well as to rat^127^ and primary human OA chondrocytes^128^ by forming cytoplasmic extensions.

In contrast, direct mitochondria delivery involves mitochondrial transplantation, typically via intra-articular injection of isolated mitochondria. In vivo, the therapeutic potential of this approach has been demonstrated in chemically-induced OA in rat models, using mitochondria isolated from rat BM-MSC or from the L6 rat myoblast cell line. These studies reported improved mitochondrial functions and attenuated OA progression.^129,130^ However, both studies lacked analysis of the distribution and kinetics of transplanted mitochondria within joint tissues.

More recently, a follow-up study using a chemically-induced OA mouse model provided additional insights.^131^ In this study, xenogeneic mitochondrial transplantation using human umbilical cord MSC (UM-MSC) showed no inflammatory response and no leakage from the knee injection site up to 24 h post-injection. Historically, stem cell therapies have been attributed a broad range of beneficial effects in OA, particularly through their immunomodulatory action. However, emerging evidence suggests that additional mitochondria-related mechanisms, such as spontaneous mitochondrial transfer from stem cells to chondrocytes, may contribute to the therapeutic effect observed in preclinical models. Despite these encouraging experimental findings, recent clinical data have tempered expectations. Notably, results from the ADIPOA2 clinical trial indicated no significant pain and functional improvement at 6 months following a single intra-articular injection.^132^ These results highlight the challenges associated with translating stem cell-based approaches into consistent clinical benefit. In parallel, stem cells have been proposed as a source of isolated mitochondria for subsequent transplantation, representing an alternative strategy aimed at directly restoring mitochondrial functions in OA chondrocytes.

In support of this concept, delivery strategies designed to enhance mitochondrial uptake have shown promising results. For example, the use of liposome-encapsulated mitochondria enhanced intra-articular delivery to chondrocytes, reduced joint inflammation and significantly improved OA histological scores (Mankin scale) in mice.^133^

Collectively, these findings indicate that mitochondrial transplantation can reduce OA-associated features in both in vitro and in vivo models. To date, no clinical trial have explored this approach in OA, although a few have been conducted in other fields particularly in cardiology^134,135^ and neurology,^136^ with only modest outcomes. Key challenges remain, including controlling the efficient cellular uptake, long-term stability, functional integration, and safety of transplanted mitochondria.

#### Transfer of highly energetic photosynthetic units

An alternative to replacing the entire mitochondria is to rescue a single important defective function of this organelle. Accordingly, a recent study investigated the use of modified high-energy photosynthetic units, derived from spinach thylakoids, to *replace* mitochondrial energy function in OA chondrocytes (Fig. 3).^137^ In vitro, these high-energy photosynthetic units were delivered to IL-1β-treated primary human chondrocytes. Upon light stimulation, this approach successfully restored the expression of anabolic factors (*COL2A1, ACAN* and *SOX9*) while concomitantly reducing the expression of catabolic factors (*MMP3*, *MMP13*, and *ADAMTS5*) bringing them to levels comparable to those observed in controls. In vivo, intra-articular injection of these high-energy photosynthetic units in a PTOA mouse model led to significant improvement, including pain alleviation and reduced cartilage degradation as reflected by lower OARSI scores.^137^

This innovative study confirms the critical role of energy production in maintaining cartilage homeostasis and underscores the potential of organelle-targeted therapies. However, it also points to the substantial challenges that remain in translating such cutting-edge biotechnology approaches into clinical practice.

#### Delivering mitochondrial “replace” therapies to chondrocytes

In the context of mitochondrial *replace* strategies, the delivery of whole mitochondria or highly energetic photosynthetic units specifically to chondrocytes presents several substantial challenges. A primary obstacle is achieving selective targeting of chondrocytes within a dense and avascular cartilage tissue. One potential approach involves functionalizing nanocarriers with cartilage or chondrocyte-affinity peptides, such as the cartilage-affinity peptide CAP (DWRVIIPPRPSA)^138^ or the chondrocytes-binding peptide WYRGRL,^139^ both initially identified through phage display screening. An alternative strategy relies on coating nanocarriers with chondrocyte-mimicking cell membranes, which has been reported to promote preferential uptake by chondrocytes. However, the relatively large size of mitochondria (typically 0.5–1 μm) poses an additional challenge, as it requires the use of large delivery vehicles capable of traversing the dense ECM. To address this issue, Kim et al. developed enlarged fusogenic liposomes coated with cationic DOTAP lipids. The positive surface charge of these liposomes facilitates the penetration of the dense and negatively charged ECM, which is rich in glycosaminoglycans.^133^ These DOTAP-coated fusogenic liposomes were shown to successfully deliver mitochondria to chondrocytes.

Taken together, these studies illustrate that multiple complementary delivery may be leveraged to overcome the structural and biological barriers associated with mitochondrial “*replace*” therapies. Careful consideration of targeting specificity, carrier size, and ECM penetration will be essential for the successful development of approaches to *replace* mitochondria in OA chondrocytes. For now, and in line with these current limitations, there is a pressing need to develop non-invasive, safe, and clinically feasible mitochondria-based therapies that can restore mitochondrial functions in OA chondrocytes.

### Mitochondrial repair strategies

As highlighted above, the clinical implementation of strategies to *replace* mitochondrial is challenging. Therefore, increasing attention has been directed toward mitochondrial *repair* approaches that aim to target and restore specific mitochondrial functions impaired in OA chondrocytes. Table 2 summarizes these *repair* strategies, encompassing both compounds that have already been evaluated in OA models and others that have not yet been tested in OA but may be amenable to repurposing for this indication. Notably, some of these compounds act on highly specific component of mitochondrial functions (e.g., cyclosporine A as an inhibitor of mPTP opening), whereas others exert broader effects by modulating multiple aspects of mitochondrial homeostasis (e.g., metformin, which affects mitochondrial dynamics and biogenesis).

**Table 2:** Mitochondria-targeting molecules in OA chondrocytes: insights from other pathophysiological contexts

	Mitochondria feature	Targeting drugs	Action	Mitochondrial effect in OA chondrocytes? *NA: Not Assessed in OA*	References
Structural	Dynamics	- Mdivi-1	- Fission reduction through DRP1 inhibition	- NA	^194^
		- P110	- Fission reduction through DRP1 inhibition	- NA	^195^
		- Metformin	- Fission reduction and fusion stimulation	- ↘DRP1 ↗MFN1/2	^155^
	Mitophagy	- Urolithin A (Mitopure)	- Mitophagy stimulation through PINK1/Parkin pathway activation	- ↗ PINK1/Parkin pathway	^196^
	Biogenesis	- AICAR	- Biogenesis stimulation through AMPK activation	- NA	^197^
		- Nobiletin	- Biogenesis stimulation through SIRT1/ PGC-1α axis activation	- NA	^154^
		- Metformin	- Biogenesis stimulation	- ↗PGC-1α	^155^
		- Liraglutide	- Biogenesis stimulation through PGC-1α activation	- NA	^162^
		- Bezafibrate	- Biogenesis stimulation through upregulation of PGC-1α and TFAM	- NA	^172^
Functional	Calcium homeostasis	- Ru360	- Mitochondrial Ca²⁺ overload regulation through MCU inhibition	- NA	^198^
		- CGP-37157	- Mitochondrial Ca²⁺ overload regulation through NCX inhibition	- NA	^199^
	Intrinsic apoptosis	- Cyclosporin A	- Mitochondria-mediated apoptosis inhibition through blockade of mPTP opening	- NA	^200^
		- Resveratrol	- Limitation of mitochondria-mediated apoptosis	- ↗ΔΨm ↘ cytochrome c release	^145^
	Redox state maintenance	- MitoQ (Mitoquinone)	- Limitation of oxidative stress through mtROS neutralization	- ↗NRF2 and GPX4	^201^
		- SS-31 (Elamipretide)	- Limitation of oxidative stress through the inhibition of mtROS production	- ↘mtROS	^202^
		- Tempol	- Limitation of oxidative stress through enhanced mtROS neutralization (SOD mimetic)	- NA	^203^
		- MnL4	- Limitation of oxidative stress through enhanced mtROS neutralization (SOD mimetic)	- NA	^204^
		- Resveratrol	- Limitation of oxidative stress through enhanced anti-oxidant systems activation	- ↘ROS	^145^
	Energy production	- Dichloroacetate or DCA	- OxPhos stimulation through PDK inhibition	- ↘Glycolytic shift	^205^
		- Nobiletin	- OxPhos stimulation through enhancing MRC expression and activity	- NA	^152^
		- Resveratrol	- OxPhos stimulation	- ↗ATP levels	^145^
		- Liraglutide	- OxPhos stimulation	- NA	^163,164^
		- Bezafibrate	- OxPhos stimulation and enhanced ATP production	- NA	^171^

Δ*Ψm* mitochondrial membrane potential, *AICAR* 5-aminoimidazole-4-carboxamide ribonucleotide, *ATP* adenosine triphosphate, *DCA* dichloroacetate, *DRP1* dynamin-related protein 1, *FIS1* mitochondrial fission 1 protein, *GPX4* glutathione peroxidase 4, *MCU* mitochondrial calcium uniporter, *MFN* mitofusin, *MRC* mitochondrial respiratory chain, *mtROS* mitochondrial reactive oxygen species, *NCX* sodium-calcium exchanger, *NRF2* nuclear respiratory factor 2, *OxPhos* oxidative phosphorylation, *Parkin* E3 ubiquitin-protein ligase Parkin, *PDK* pyruvate dehydrogenase kinase, *PGC-1α* peroxisome proliferator-activated receptor gamma coactivator 1 alpha, *PINK1* PTEN-induced putative protein kinase 1, *SIRT1* sirtuin 1, *SO2* superoxide dismutase 2, *TFAM* transcription factor A mitochondrial

Among the various mitochondrial functions, energy metabolism appears to be central. Both glycolysis inhibition (a mitochondria independent process) and OxPhos stimulation (a mitochondria dependent process) have therefore attracted interest. For example, glycolysis inhibition by replacing glucose with galactose or 2-deoxy-d-glucose (2-DG), a non-metabolizable glucose analog, has been shown to reduce the expression of catabolic markers in OA chondrocytes.^32,42^ Additionally, pharmacological inhibition of glycolytic enzymes, such as LDHA with oxamate, has demonstrated similar effects.^140,141^ However, a complete inhibition of glycolysis can compromise essential chondrocyte cellular functions, highlighting the need for alternative strategies that promote OxPhos rather than inhibit glycolysis.

Addressing OxPhos dysfunction in OA is inherently complex and raises seemingly ambivalent therapeutic considerations. On one hand, partial inhibition of mitochondrial respiration may be beneficial by limiting excessive superoxide generation, which contributes to oxidative stress and inflammation. For instance, in an in vitro experimental OA chondrocyte model using immature chondrocytes treated with IL-1β, the pharmacological inhibition of the MRC with rotenone or antimycin A, reduced *IL6* and *MMP13* gene expression, as well as the protein levels of the inflammatory mediator IκB-ζ.^142^ Similarly, in a porcine hock intra-articular fracture model of OA, the administration of amobarbital (a complex I inhibitor) suppressed OxPhos, thereby reducing mitochondrial superoxide production and oxidative stress. It conferred protection against OA development, as reflected by the reduction of the Mankin histological scores.^143^

However, it is important to note that these experimental settings predominantly reflect acute stress responses rather than the chronic mitochondrial alterations characteristic of OA chondrocytes in vivo. In contrast, the majority of studies have focused on strategies aimed at enhancing OxPhos in order to improve ATP production and support anabolic process in cartilage. In this context, most evidence support the interest of promoting OxPhos. Notably, *Pdk2* KO mice, which favor the conversion of pyruvate to acetyl-CoA over lactate, thereby enhancing OxPhos, have shown resistance to cartilage damage and pain in PTOA mouse models.^144^ Overall, this finding supports the idea that targeting energy metabolism dysfunction, either directly or indirectly by stimulating mitochondrial biogenesis to improve mitochondrial number, is a relevant therapeutic strategy for OA.

Among the various therapeutic interventions, small molecules designed to restore specific mitochondrial functions are emerging as particularly attractive candidates. In the following section, we place special emphasis on compounds that enhance mitochondrial energy metabolism, especially those with the additional capacity to ameliorate other aspects of mitochondrial dysfunctions. The following sections therefore focus on these mitochondrial *repair* approaches, illustrating them with several cases, including both already assessed and innovative molecules with potential relevance for OA treatment.

#### Flavonoids

Flavonoids, a subgroup of polyphenolic bioactive natural compounds, have been extensively studied in OA, mainly for their anti-inflammatory properties. Interestingly, as other polyphenolic compounds, flavonoids can also exert beneficial effects on mitochondrial functions. The following part focuses on the role of flavonoids as in mitochondria *repair* approaches, using two representative examples that range from an extensively studied compound (Resveratrol) to a newly suggested candidate (Nobiletin).

##### Resveratrol

Among flavonoids, resveratrol is of particular interest in OA. In vitro studies using primary human OA chondrocytes have demonstrated that resveratrol can reverse IL-1β-induced mitochondrial defects by restoring mitochondrial membrane potential (Δ*Ψm*), ATP production and mitochondrial morphology, while simultaneously reducing cytochrome c release and ROS production (Fig. 3).^145,146^ Additionally, resveratrol exhibits anti-inflammatory and anti-catabolic properties, notably by inhibiting cyclooxygenase 2 (COX-2) activity and reducing the production of prostaglandin E2 (PGE2), and MMP-13 in OA chondrocytes.^145^ In vivo, both intra-articular and intraperitoneal administration of resveratrol have been shown to attenuate the progression and severity of PTOA in rats and mouse models.^147–150^ However, despite these promising preclinical results, a recent phase III clinical trial (NCT02905799) evaluating oral resveratrol supplementation in patients with knee OA failed to demonstrate significant pain relief as the primary clinical outcome, compared with placebo.^151^ This discrepancy between preclinical efficacy and clinical outcome may, at least in part, reflect the pleiotropic and non-specific nature of resveratrol effects on mitochondrial and cellular pathways, which may limit its translational relevance in OA.

##### Nobiletin

Beyond resveratrol, other flavonoids, which have been previously investigated in the context of neurodegenerative and metabolic diseases, may offer therapeutic potential for OA, notably through their effects on mitochondrial functions. Nobiletin is a flavonoid compound that has been shown to enhance mitochondrial energy metabolism by upregulating the expression and activity of MRC complexes I and II, thereby improving the overall mitochondrial respiration in skeletal muscle cells^152^ and cortical neurons.^153^ Additionally, nobiletin has been found to stimulate mitochondrial biogenesis, as evidenced by increased expression of key regulators such as SIRT1 and PGC-1α in studies involving in vitro oocytes maturation.^154^ Across these various conditions, nobiletin consistently reduces ROS levels and enhances antioxidant defense mechanisms, supporting its potential relevance for mitigating mitochondrial dysfunctions in OA.

#### Repurposing clinically approved drugs

The repurposing of clinically approved drugs represents another therapeutic strategy to modulate mitochondrial activity in the context of OA. Building of the previous section, this part focuses on clinically approved drugs that have already been studied for repurposing in OA such as metformin and liraglutide, and introduces bezafibrate as a newly suggested candidate for mitochondria *repair* strategies.

##### Metformin

One such example is metformin, an FDA-approved anti-diabetic drug. A study published in 2019 demonstrated that metformin exerts a promising effect on IL-1β-treated murine chondrocytes by reducing MMP13 protein levels and oxidative stress, while increasing mitochondrial membrane potential and COL2A1 protein levels. Moreover, metformin upregulates PGC-1α protein expression levels, promotes mitochondrial fusion through increased expression of MFN1/2, and inhibits fission via the downregulation of DRP1. This study also showed that metformin can activate the SIRT3/PINK1/Parkin-mediated mitophagy pathway. Collectively, these data suggest that metformin may counteract multiple aspects of mitochondrial dysfunctions in OA chondrocytes (Fig. 3).^155^ Notably, intra-articular administration of metformin in PTOA mouse models has been shown to significantly ameliorate OARSI scores.^156^ From a translational perspective, at least two clinical trials have reported beneficial effects of metformin on pain outcomes in patients suffering from knee OA.^157,158^ In addition, eight other clinical trials registered on ClinicalTrials.gov (NCT06126029, NCT06231758, NCT07065591, NCT06328426, NCT05034029, NCT06367283, NCT06096259, NCT05638893) are currently ongoing or have been completed to evaluate the effect of metformin in OA, although their results have not yet been published.

##### Liraglutide

Other FDA-approved anti-diabetic drugs may also hold the potential to modulate mitochondrial function. One notable example is liraglutide (commercially known as Victoza®), a glucagon-like-peptide-1 (GLP-1) analog that has shown beneficial effects in the context of OA. Intra-articular injection of liraglutide significantly reduces pain-related behavior in a chemically-induced OA mouse model. Liraglutide also downregulates the expression of catabolic genes (*MMP3*, *MMP13*, *ADAMTS4*, *ADAMTS5*) and inflammatory genes (*TNF-α*, *COX-2*) in IL-1β-treated murine chondrocytes^159^ as well as in OA rat models in vivo.^160^ Beyond OA-related models, liraglutide has been shown to promote mitochondrial biogenesis in human adipocytes^161^ and in mouse models of Parkinson’s disease, notably through activation of the PGC-1α pathway.^162^ Liraglutide also reduces oxidative stress, promotes mitochondrial fusion and improves energy metabolism in various murine disease models.^163,164^ However, in a randomized clinical trial investigating the effect of liraglutide in knee OA (NCT02905864), no significant reduction in knee pain was observed compared with placebo.^165^ There is still a growing interest in targeting the GLP-1/GLP-1 R axis in OA research,^166,167^ and other GLP-1 analogs, such as exendin or semaglutide, are emerging as potential candidates. Nonetheless, the mechanisms by which these molecules influence mitochondrial dysfunctions in the context of OA remain largely uncharacterized and further studies are needed to better understand these mechanisms.

##### Bezafibrate

Repurposing clinically approved drugs with known effects on mitochondrial functions that have not yet been studied in the context of OA is particularly encouraging. One promising candidate is bezafibrate, a pan-peroxisome proliferator-activated receptor (PPAR) agonist currently approved for dyslipidemia treatment. Interestingly, bezafibrate belongs to the fibrate family of PPAR agonizts, which is relevant in the context of OA. Indeed, another member of this family, fenofibrate, has already been shown to regulate key OA-related mechanisms such as senescence and autophagy.^168^

Bezafibrate has been shown to improve mitochondrial biogenesis in various disease models, including a rat model of neurometabolic disease^169^ and multiple mouse models.^170^ Additionally, bezafibrate significantly increases ATP production in OxPhos-deficient fibroblasts.^171^ Moreover, in neural progenitor cells, bezafibrate reduces ROS levels and promotes mitochondrial biogenesis by upregulating PGC-1α (*PPARPGC1A* gene) and TFAM expression, as well as enhancing mtDNA copy numbers.^172^ Collectively, these findings support bezafibrate as a promising candidate for targeting mitochondrial dysfunctions in OA chondrocytes and warrant further investigation in preclinical OA models.

Some mitochondrial-*repair-*oriented therapeutic molecules may benefit from direct mitochondrial targeting to maximize their efficacy. Accordingly, such active compounds could be conjugated to functionalized nanocarriers previously defined, through established mitochondrial-targeting motifs. This includes lipophilic cations such as triphenylphosphonium (TPP) or dequalinium (DQA), as well as mitochondria-targeting peptides, notably the Szeto-Schiller (SS) peptide, among which SS-31 (elamipretide) is the most extensively characterized.^173^ A comprehensive overview of these compounds has been provided in a recent review.^174^ Nevertheless, it is important to note that many candidates for mitochondrial-*repair* therapies for OA chondrocytes may also exert beneficial effects indirectly. In particular, such compounds can modulate the transcription of nuclear-encoded genes that regulate mitochondrial function, biogenesis, or dynamics, thereby improving mitochondrial homeostasis without requiring direct mitochondrial targeting.

## Conclusion and future directions

Mitochondria play a pivotal role in maintaining chondrocyte homeostasis through their various functions, including energy production, redox balance, and regulation of apoptosis. Under pathological conditions, these functions are frequently disrupted and are now well established to contribute to key OA features such as inflammation and oxidative stress, two processes that have been targeted by previous therapies with limited benefits.

These observations highlight an urgent need to develop novel therapeutic strategies that directly address mitochondrial dysfunctions of OA chondrocytes as a critical component of OA pathogenesis. Mitochondrial dysfunctions are increasingly recognized as a therapeutic target in various diseases and may hold similar potential in OA. Recent preclinical studies involving mitochondrial transplantation have paved the way for the development of mitochondrial-based interventions in OA. However, despite their conceptual appeal, substantial technical and translational challenges still remain to be overcome before intra-articular mitochondrial transplantation can be realistically envisioned for OA treatment. These challenges include methods for delivering mitochondria to avascular cartilage in a tissue- or cell-specific manner, ensuring the persistence of therapeutic effects, and managing the safety and risks associated with the required repeated intra-articular injections.

Looking beyond chondrocytes, evidence, although still limited, suggests that mitochondrial alterations may also affect other joint-resident cells. In synovial tissue, some studies report mitochondrial alterations in both FLS and macrophages. Similar to OA chondrocytes, OA-FLS display impaired OxPhos, metabolic shift toward glycolysis, increased mtROS production, and dysregulated apoptosis.^175–177^ More recent work has expanded this mitochondrial dysfunctions phenotype, revealing increased mitochondrial fission, reduced mitochondrial membrane potential, and decreased cristae density.^177^ Interestingly, the glycolytic reprogramming of OA-FLS can promote macrophages polarization toward the pro-inflammatory M1 phenotype, which is itself characterized by high glycolytic flux and low OxPhos activity.^178–181^

In contrast, direct evidence of mitochondrial dysfunctions in bone cells during OA remains scarce. However, altered mitochondrial dynamics and mitochondrial-related oxidative stress in bone profoundly influence its remodeling in other pathological contexts.^182–184^ These observations suggest that mitochondrial alterations in bone cells may contribute to abnormal bone structure in OA, potentially promoting pathological bone features such as osteophyte formation.

Overall, the description of mitochondrial dysfunctions in synovial and bone cells during OA is still limited and fragmented, underscoring the need for further experimental validation. Expanding mitochondrial-focused investigations in these cells, as well as to additional cell types, including infrapatellar fat pad adipocytes, ligamentocytes, and a broader spectrum of synovial immune cells, represents a critical unmet need for a comprehensive understanding of OA pathophysiology with respect to mitochondrial dysfunctions.

In light of the current technical constraints surrounding mitochondrial transplantation and the limited knowledge of mitochondrial dysfunctions across joint tissues, future research efforts should prioritize alternative strategies aimed at restoring mitochondrial functions in OA chondrocytes. In this context, the repurposing of innovative mitochondria-targeting molecules already under clinical or preclinical investigation for other diseases represents a pragmatic and promising avenue. Further rigorous studies are now needed to assess their efficacy, safety, and therapeutic relevance in OA to translate mitochondrial biology into meaningful disease-modifying interventions.
